# Comparison of CPAP with Humidifier, Blender, and T-piece on the Outcome of Weaning in Patients with Neurological Disorders

**Published:** 2015

**Authors:** Nemat BILAN, Shalaleh GANJI

**Affiliations:** 1 Pedaitric Polmonologist. Pediatric Health Research Center, Tabriz University of Medical Sciences,Tabriz, Iran; 2 Pediatrics Department, Tabriz University of Medical Sciences,Tabriz, Iran

**Keywords:** CPAP, T-piece, Blender, Humidifier, Neurological Disorder

## Abstract

**Objective:**

The procedure for weaning from mechanical ventilation in many patients is a difficult and long process and increases the time of mechanical ventilation. There are numerous ways to achieve weaning. One common method is the use of CPAP. Considering the lower price of a humidifier, blender, and T-piece compared with CPAP and in light of the limited number of studies in this field, the current study purposed to compare these two procedures.

**Materials & Methods:**

Fifty-one patients with neurological disorders who were under mechanical ventilation and ready to wean were allocated randomly into two groups: the CPAP group and the humidifier, blender, and T-piece group. Duration of hospital and PICU stay, number of days under mechanical ventilation, frequency of reintubation, and mortality rate among patients were documented.

**Results:**

The patients were 33 males and 18 females (64.7% and 35.3%, respectively) with an average age of 22.5 ± 4.5 months. The main indication for intubation was impending respiratory failure. Hospital stay was 22±15 and 21±13 days for the humidifier and CPAP groups, respectively. PICU stay was 13±11 and 21±13 days for the humidifier and CPAP groups, respectively. Re-intubation rates were 17.2% and 45.5% for the humidifier and CPAP groups, respectively. Mortality rates were 3.4% and 22.5% for the humidifier and CPAP groups, respectively.

**Conclusion:**

Considering no statistically significant difference between the two groups, using the humidifier, blender, and T-piece is recommended.

## Introduction

Acute lung injury and acute respiratory failure are the main causes for applying mechanical ventilation in children in order to help them survive. Weaning patients from mechanical ventilation has a significant importance in the treatment course and outcome of these patients. Weaning is the procedure through which patients are gradually separated from the mechanical ventilator. In fact, it is the phase in which the act of breathing is transferred from the ventilator to the patient. This procedure is usually not easily done in patients with an acute episode of respiratory failure. It is a long and difficult process in many patients and consumes a considerable amount of health system resources. This process includes almost 40% of total mechanical ventilation (1 and 2). One method for weaning patients from the ventilator is the spontaneous breathing trial (3). This trial is usually conducted with a T-piece and/or an instrument for oxygen transference through a trachestomy and/or with positive pressure (CPAP). The T-piece requires a high gas pressure; the other applied instrument is a machine which combines the temperature and humidity with air (4). Currently, there are two hospital machines used for making patients’ inhalation warm and humid during endotracheal intubation and for compensating for nasal function (bypass intubation) (5). In the last few years numerous studies have been conducted on limiting ventilation time by early identification of those patients eligible for being weaned from the ventilator, designing methods for testing the patient’s readiness for weaning from the ventilator and continuing spontaneous breathing, and methods for reducing support for patients who have been unsuccessful in spontaneous breathing trials (6-9). In recent years, the main concern for physicians has been separating patients from mechanical ventilation and reducing the ventilation period with the least possible side effects. It has been clearly recognized that when a patient is intubated, the heat and humidity exchanging process of breathing gases, which is performed by the upper airways, is bypassed, because the respiratory airways are omitted from the respiration course. The lack of sufficient humidity may cause a decrease in the coughing reflex, an increase in bronchial discharges and, as a consequence, an increase in the number and duration of suction times. A decrease in mucociliary clearance, the destruction of respiratory cilia and mucous glands, alterations in lung functions, and a decrease in the body’s central temperature are other adverse effects of insufficient humidity and heat in airways (11-14). In order to assess a patient’s respiratory system and breathing ability at the time of weaning from mechanical ventilation, the spontaneous breathing trial is often performed, usually with the use of a T-piece, humidifier, and blender (15). In a study by Jones et al., the two procdures of humidifier, blender, and T-piece and CPAP in weaning adult patients from mechnical ventilation were compared to mechanical intubation, and results showed the two groups were identical. No difference was observed in heartbeat or systolic and diastolic blood pressure between the two groups (16). Jones et al. concluded that the use of the humidifier, blender, and T-piece caused no disturbance in arterial oxygenation and, in fact, may be preferred to using CPAP. In another study by Molina-Saldarriaga et al., the use of the T-piece was compared with CPAP in weaning patients from the humidifier, blender, and T-piece. The rates of successful weaning of patients from mechanical ventilation in the two groups were 60% and 76%, respectively. Contrary to the results obtained from previous studies (17), Molina-Saldarriaga et al. reported that the use of CPAP achieved more satisfactory results, although more studies are needed to confirm this result. In a similar study by Vats et al., from the 20 patients in the humidifier, blender, and T-piece group, 15 patients were extubated, of which 5 patients needed reintubation. Among the 20 patients in the CPAP group, 17 patients were extubated, of which only 3 required re-intubation. Even with this difference, Vats et al. concluded that the importance and effectiveness of these two methods in weaning patients from mechanical ventilation are equal (18). In their study, Estaban et al. also found these two methods to be of equal importance. In other studies, the effects of using different warming and humidifying machines in noninvasive ventilation were compared. In one of these studies, Lellouche et al. compared the effects of warmth and humidity interchange using the two methods of end-tidal positive pressure and T-piece (19). In a similar study, Jaber et al. showed that PaCo2 was higher when a warmth and humidity exchanger was used in comparison with warm humidifiers, while the ratio of PaO2 to FiO2 was not significantly different (20). Numerous studies have been conducted on the effects of making the air flow warm or humid during treatment, but none of them focused on patients with neuromuscular disorders; therefore, this study was conducted.

## Materials & Methods

This study was a randomized clinical trial (N1IRCT 2013043013189). Enrolled in the study were 51 patients with neurological disorders who were hospitalized in the PICU of Tabriz Children’s Hospital and underwent mechanical ventilation. Patient information was totally confidential, and the parents of all participants read and signed an informed consent form. They were also reassured that they could withdraw from the study at any time. At the time of weaning, patients were randomly assigned into either the CPAP group or the humidifier, blender, and T-piece group. Patients who had spontaneous respiration and received less than 40% FiO2 with PaO2 of more than 60% were considered candidates for weaning from the ventilator. During hospitalization, the number of days under mechanical ventilation, the need for re-intubation, the number of days hospitalized, and the number of days in PICU were documented for each patient. Descriptive statistics (frequency, percentage, and average± normal deviation) were used for statistical investigations. To compare the qualitative findings, the statistical test of χ2 was used, and to compare quantitative findings between groups, the independent t-test was used. A p value less than 0.05 was considered significant.

## Results

**Fig 1:**
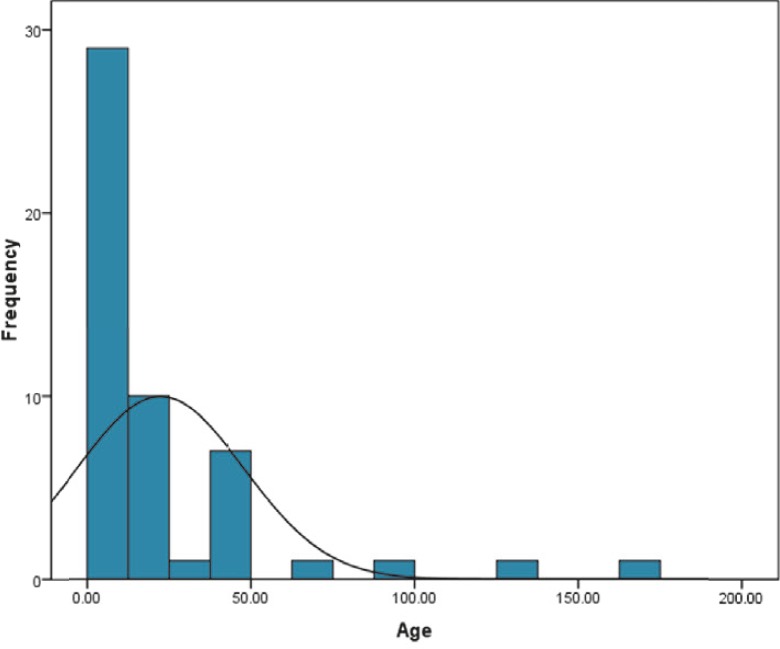
Distribution of patient’s ages

Participants in this study included 33 male (64.7%) and 18 female patients (35.3%). Average patient age was 22.5 ± 4.5 months. Minimum and maximum ages of the patients were 1.5 and 164 months, respectively. The median and the mode were 10 and 48 months, respectively (Figure 1). Hospitalization in PICU was indicated by extensive respiratory distress in a significant number of patients, and by cardiopulmonary arrest and reduced consciousness in others.

The main indication for intubation was impending respiratory failure. Hospital stay was 22±15 and 21±13 days in the humidifier and CPAP groups, respectively (p=0.48). PICU stay was 13±11 and 21±13 days in the humidifier and CPAP groups, respectively (p=0.16). Re-intubation rates were 17.2% and 45.5% in the humidifier and CPAP groups, respectively (p=0.1). Mortality rates were 3.4% and 22.5% in the humidifier and CPAP groups, respectively (p=0.07).

## Discussion

Weaning patients from mechanical ventilation is a vital part of caring for intubated patients. There is no global agreement on the best method for this process. Considering the lack of studies comparing different methods for weaning child patients, the need for such studies in the form of clinical trials is significant. In the current research, the effectiveness of the humidifier, blender, and T-piece in weaning patients from mechanical ventilation was studied. Fifty-one patients with neurological disorders who were under mechanical ventilation and showed indications for weaning from it were randomly assigned to two study groups. As was expected, in this study like in others, pneumonia was the main cause of acute respiratory failure (10). As mentioned before, there has been no study in which children were considered. For this reason, it is not possible to compare the results of the basic study with other studies. Causes of respiratory failure are different in different age groups. Extensive respiratory distress was the indication for hospitalization in the ICU for a considerable number of patients. The main cause for patient intubation was impending respiratory failure. What is clear is the considerable difference in causes of respiratory failure and the need for mechanical ventilation in children compared to adults, which is a reminder of the importance of studying these two age groups separately (16-20). Considering this fact, the presence of considerable effects of the humidifier, blender, and T-piece in reducing the number of re-intubation cases can be promising in reducing the incidence rate of mortality in patients with acute respiratory failure. Conclusion: Considering no statistically significant differences between the two groups, use of the humidifier, blender, and T-piece is recommended.
